# Past, Present, and Future of Blood Biomarkers for the Diagnosis of Acute Myocardial Infarction—Promises and Challenges

**DOI:** 10.3390/diagnostics11050881

**Published:** 2021-05-15

**Authors:** Ioan Tilea, Andreea Varga, Razvan Constantin Serban

**Affiliations:** 1 Department M4, Clinical Sciences, Faculty of Medicine, “G. E. Palade” University of Medicine, Pharmacy, Science and Technology of Targu Mures, 540142 Targu Mures, Romania; ioan.tilea@umfst.ro; 2 Department of Cardiology II, Emergency Clinical County Hospital, 540042 Targu Mures, Romania; 3 Department ME2, Faculty of Medicine in English, “G. E. Palade” University of Medicine, Pharmacy, Science and Technology of Targu Mures, 540142 Targu Mures, Romania; 4 Cardiac Catheterization Laboratory, The Emergency Institute for Cardiovascular Diseases and Transplantation, 540136 Targu Mures, Romania; serbanrazvan1@gmail.com

**Keywords:** acute myocardial infarction, blood biomarkers, diagnosis

## Abstract

Despite important advancements in acute myocardial infarction (AMI) management, it continues to represent a leading cause of mortality worldwide. Fast and reliable AMI diagnosis can significantly reduce mortality in this high-risk population. Diagnosis of AMI has relied on biomarker evaluation for more than 50 years. The upturn of high-sensitivity cardiac troponin testing provided extremely sensitive means to detect cardiac myocyte necrosis, but this increased sensitivity came at the cost of a decrease in diagnostic specificity. In addition, although cardiac troponins increase relatively early after the onset of AMI, they still leave a time gap between the onset of myocardial ischemia and our ability to detect it, thus precluding very early management of AMI. Newer biomarkers detected in processes such as inflammation, neurohormonal activation, or myocardial stress occur much earlier than myocyte necrosis and the diagnostic rise of cardiac troponins, allowing us to expand biomarker research in these areas. Increased understanding of the complex AMI pathophysiology has spurred the search of new biomarkers that could overcome these shortcomings, whereas multi-omic and multi-biomarker approaches promise to be game changers in AMI biomarker assessment. In this review, we discuss the evolution, current application, and emerging blood biomarkers for the diagnosis of AMI; we address their advantages and promises to improve patient care, as well as their challenges, limitations, and technical and diagnostic pitfalls. Questions that remain to be answered and hotspots for future research are also emphasized.

## 1. Introduction

Cardiovascular diseases are responsible for almost half of all fatalities worldwide, causing more than four million deaths each year in Europe alone [1]. Among these, coronary artery disease is the leading cause of death, with acute myocardial infarction (AMI) accounting for most of the mortality related to coronary artery disease [2]. Changes in human lifestyle and behavior, particularly in developing countries, have led to a continuous, rapid increase in AMI incidence over recent decades, with annual growth rates of more than 3.5% [3]. About 10% of patients who present to emergency departments with chest pain every year are diagnosed with AMI [4].

Recognition that AMI most commonly occurs as a result of intracoronary thrombosis and that early opening of the occluded coronary artery significantly ameliorates outcomes in this high-risk population has reshaped AMI diagnosis over the years. Major interest emerged in developing strategies that would allow both early recognition and exclusion of AMI. The former would enable rapid, often life-saving interventions; the latter would allow rapid and safe patient discharge, considerably reducing healthcare costs. Diagnosis of AMI continues to be mainly driven by the occurrence of acute chest pain in the presence of typical ECG changes. However, approximately 90% of patients presenting with chest pain do not have AMI, and the sensitivity and specificity of ECG changes in AMI are rather low [4,5]. Moreover, there is a small, although non-negligible, proportion of patients that do not exhibit obvious symptoms and/or ECG changes. This context has emphasized the need for additional diagnostic criteria, and cardiac biomarkers have emerged as the most obvious approach.

**Figure 1:**
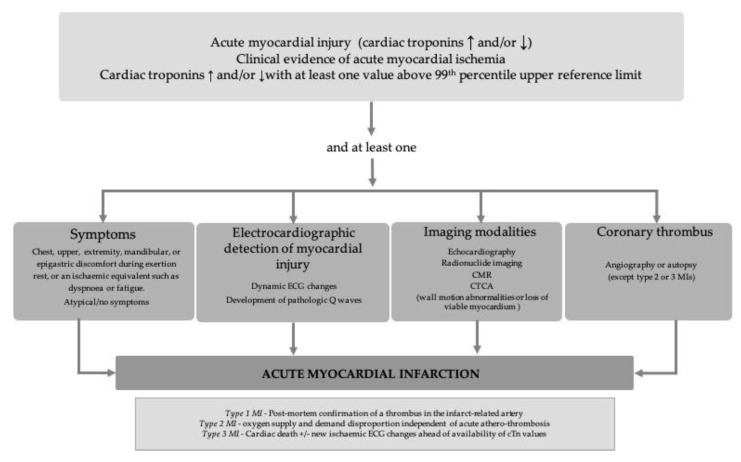
Schematic diagram of the universal definition of acute myocardial infarction, adapted from [7]: CMR—cardiac magnetic resonance; CTCA—computed tomographic angiography; MI—myocardial infarction; cTn—cardiac troponins.

Initially, serial, daily measurement of cardiac biomarkers served only as a strategy to retrospectively confirm AMI diagnosis. Since then, their role has become increasingly larger, and cardiac biomarker changes are now included as major diagnostic criteria in AMI (Figure 1) [6].

This article reviews the evolution, current application, and emerging biomarkers for the diagnosis of AMI, addressing their advantages and promises to improve patient care, as well as their challenges, limitations, and technical and diagnostic pitfalls. Questions that remain to be answered and hotspots for future research are also emphasized.

## 2. Out-of-Date Biomarkers for Acute Myocardial Infarction Diagnosis

Severe myocardial ischemia and the consequent myocardial necrosis lead to the release of a plethora of cardiac enzymes into the circulation. Thus, markers such as myoglobin, lactate dehydrogenase (LDH), aspartate aminotransferase (AST), and creatine kinase (CK), including the concept of delta change, were introduced as initial indicators of AMI. Historically, AST and LDH were the first cardiac enzymes used for AMI diagnosis [8].

### 2.1. Aspartate Aminotransferase

Aspartate aminotransferase is a ubiquitous, soluble, intracellular enzyme critical in amino acid metabolism. The largest amounts of AST are expressed in the liver, the myocardium, the kidney, and the skeletal muscle. In 1954, Ladue et al. demonstrated a significant rise in AST 3–4 h after an AMI, beginning the age of enzyme-based AMI diagnosis [9]. Blood levels of AST increase within the first 12–24 h after AMI, reach a peak 1–2 days after the acute event, and return to baseline within 10–14 days after AMI (Table 1).

The ubiquitous expression of AST in a wide variety of tissues significantly affects its specificity for myocardial injury, limiting its use as a cardiac biomarker. Currently, AST is no longer used for AMI diagnosis.

### 2.2. Lactate Dehydrogenase

Only one year after the advent of AST as an AMI biomarker, LDH, an enzyme that reversibly converts lactate to pyruvate, emerged as a new promising indicator of AMI [10]. Blood levels of LDH typically increase within 6–12 h after the onset of AMI, peak within the following 1 to 3 days, and return to baseline within 8–14 days (Table 1). Similarly to AST, LDH is also expressed in a wide variety of tissues, including the liver, the kidney, the heart, the red blood cells, the lung, and particularly, the skeletal muscle, which makes LDH a marker with poor specificity for cardiac injury (Table 2).

Of the five LDH izoenzymes, the heart expresses LDH-1 with four heart subunits (H_4_); the isomeric form LDH-2 exists in a tetrameric combination of three heart and one muscle subunit (H_3_M_1_) [11]. Hence, an LDH-1/LDH-2 ratio >1 has been proposed as a specific AMI marker. The LDH-1 isoenzyme is not highly specific to the heart, however, and the LDH-1/LDH-2 ratio did not impose as a relevant AMI biomarker [12].

Nowadays, the only use of LDH is in distinguishing between acute and subacute AMI in the late stage of the ischemic event when other cardiac markers have already returned to their normal levels.

### 2.3. Myoglobin

Myoglobin is a low molecular weight iron- and oxygen-binding protein abundantly expressed in the myocardium and skeletal muscle. Myoglobin is rapidly released by the injured myocardium. Its blood levels start to increase within the first 30 min to 2 h after the onset of ischemia, which makes myoglobin an important marker for the early detection/exclusion of cardiac injury. Its levels increase during the first 6–10 h after AMI, reach a peak ≈12 h after the acute event, and return to baseline by 24 h after AMI (Table 1). Myoglobin is not found in any other tissue than the muscle, making it a sensitive marker for AMI, with high negative predictive value, and therefore is a useful test to rapidly rule out AMI in the emergency room. However, because myoglobin expression is not restricted to the myocardium (Table 2), its specificity and positive predictive value are rather low [13].

### 2.4. Creatine Kinase and Creatine Kinase Myocardial Band

Creatine kinase is an enzyme abundantly expressed in the myocardial cells, where it catalyzes reversible transfer of high-energy phosphate from ATP to creatine, producing creatine phosphate. Since the early 1970s, and particularly since the 1980s, with the advent of the ELISA technique, CK has become a crucial laboratory parameter for the identification of myocardial damage and AMI. The enzyme is present, however, in a large variety of other tissues (Table 2), strongly affecting its specificity as a biomarker of myocardial damage. This issue has been partly overcome by use of the CK-myocardial band (CK-MB) isoform, found in the heart, where it represents ≈20% of total CK, but also in the skeletal muscle, diaphragm, uterus, and several other issues [14]. With ≈91% sensitivity and specificity for the diagnosis of AMI during the first 6 h after symptoms onset, CK-MB has become widely used in emergency department settings [15]. Its levels start to increase 4–9 h after the onset of AMI, reach a peak within the first 24 h, and return to baseline during the next 48–72 h (Table 1). In patients with AMI, there is a strong correlation between CK and CK-MB levels and infarct size, making these markers suitable for estimating the severity of AMI. Evaluation of CK-MB has, however, several major drawbacks. Firstly, due to its high molecular weight, CK-MB has limited ability to detect minor myocardial damage. Secondly, CK and CK-MB expression is not restricted to the heart. Hence, a number of other conditions can also lead to significant CK and CK-MB increases (Table 2), reducing their diagnostic specificity for AMI. Thirdly, cross reactivity can occur with other different compounds, including heterophilic antibodies such as rheumatoid factor. Due to these flaws, CK-MB evaluation is no longer recommended for AMI diagnosis, although it still has a role in estimating infarct size [7,16].

## 3. Cardiac Troponins—The Current Gold Standard in the Laboratory Diagnosis of Acute Myocardial Infarction

Along with the introduction of new myocardial injury biomarkers, the sensitivity for the diagnosis of AMI also recorded an important rise. However, because all previously identified markers were also expressed in a variety of other tissues, specificity continued to be problematic (see Table 2). Hence, numerous patients presenting with non-AMI and even non-cardiac chest pain syndromes continued to undergo extensive, costly, and often invasive evaluations. At the same time, traditional enzyme evaluation was largely incapable of detecting AMI patients with small amounts of cellular death. Introduction of cardiac troponins (cTn) as biomarkers of myocardial injury has radically changed the landscape of AMI diagnosis. At present, cTn are the most widely used, evidence-based, and guideline-endorsed biomarkers of AMI, manifesting a major and immediate impact on AMI patients’ management [16].

Nevertheless, values above the upper reference limit of cardiac troponins can be detected in other circumstances unrelated to a thrombotic acute coronary event [17].

Patients with moderate chronic kidney disease or dialyzed patients present persistent elevated cTn values, associated with increased cardiac death and all-cause mortality [18]. In clinical and ECG features suggestive for an AMI, in an end-stage chronic kidney disease patient, dynamic changes higher or equal to 20% in cTn over 6 to 9 h should be interpreted as positive for an AMI [17].

However, the prognostic role of cardiac troponins in the assessment of other potential causes of elevation showed an important independent value, such as in acute heart failure. Serial measurements of troponin can be used for risk assessment in heart failure out-patients, because regular or persistent elevations of cTn are corelated with high risk of death or hospital readmission [19]. The cut off values of troponin T in predicting mortality in acute heart failure is variable in studies with different endpoints, varying from 0.01 ng/mL to cTn above URL or the 99th percentile. [20]

Elevated plasma troponin levels in acute phase of pulmonary embolism are defined as concentrations above upper reference limits; these limits are assay dependent [21].

The rule-in or rule-out of AMI diagnosis based entirely on positive values of troponins addresses challenges, and these values should be interpreted considering clinical presentation, serial ECGs, and other patient variables.

In the myocardium, troponin exists as a hetero-trimer composed of troponin I (TnI), T (TnT), and C (TnC) as subunits. The troponin complex interacts with tropomyosin as part of the thin filaments of the cardiac sarcomere, regulating the calcium-dependent interaction of actin and myosin in response to cytosolic calcium changes. TnC also exists in the striated muscle, rendering it unsuitable for AMI diagnosis, whereas TnT and TnI are specific to the heart and are therefore termed “cardiac troponins”. Although TnI assays have generally been affected by more technical problems, this isoform has been shown to have greater early diagnostic accuracy and to be more specific for myocardial injury than TnT, which has also been shown to increase in settings such as skeletal muscle injury, kidney disease, malignancy, or sepsis [22,23]. In average, the amount of cTn per gram of myocardium is ≈13–15-fold higher than that of CK-MB, explaining the higher sensitivity of cTn in detecting early and/or minor myocardial damage [14]. Myocardial cells possess very small pools of cytosolic troponin, whereas the troponin bulk is located within the contractile apparatus of these cells. In contrast, circulating troponin levels are extremely low in the healthy individual. Hence, troponin plasma levels allow easy identification of even small myocyte injury. In patients with AMI, troponin release occurs initially from the cytosolic pool and later from the contractile apparatus of the damaged cells. This specific dynamic of release explains why cTn rises rapidly after the onset of myocardial ischemia, reaches a peak within the first 12–24 h, and remains high for 1–3 weeks after the acute event (Table 1).

With the advent of high-sensitivity cTn (hs-cTn) assays, sensitivity has become even higher, enabling the detection of troponin levels 10-fold lower than the initial values [24]. By definition, these assays must have an imprecision of less than 10% at the 99th percentile of a reference population and be able to measure cTn levels in at least 50% of a healthy reference population, although the most recent assays have been shown to detect troponin in >95% of healthy reference cohorts and to have an imprecision level in the range of 2–5% [16,25,26]. Overall, the added value of hs-cTn assays is not to identify more AMI cases, but rather to identify AMI more rapidly and to more quickly exclude those patients that do not have AMI [27]. Current rule-in and rule-out diagnostic algorithms rely on very low cTn cut-offs at the first presentation, and rapid, sequential measurements on admission and shortly (1–2 h) after, together with the calculation of delta troponin (i.e., rate of troponin change) [28]. Table 3 and Table 4 present the specific cut-off levels of different hs-cTn T and hs-cTn I used in the current diagnosis of myocardial infarction, by each manufacturer.

Mortality attributable to AMI has significantly declined over the years, mainly due to early recognition and rapid, effective, myocardial revascularization. Repeated testing by principal laboratories is a significant logistic challenge, however, that cannot be overcome by many of the diagnostic laboratories. Implementation of algorithms requiring fast decision-making thus had to be paralleled by earlier access to biomarkers, particularly troponin. Point-of-care tests (POCTs) for cTnI with diagnostic sensitivity comparable to that of central laboratory testing have become available, allowing markedly improved turnaround times and immediacy of results, along with improved therapeutic decision-making, patient flow and experience, and reduced costs [26]. Currently available hs-cTn POCT systems only provide diagnostic sensitivity comparable to that of central laboratory testing ≈6 h after admission, thus limiting their use. Randomized trials on the impact of POCTs in patients presenting with chest pain have also provided rather inconsistent results. However, in general, POCTs appeared to improve patient flow and decrease the length of stay in the emergency departments, while increasing hospital and coronary care unit admissions [26].

## 4. Newer Biomarkers for Laboratory Diagnosis of Acute Myocardial Infarction

The introduction of cTn testing has dramatically changed the diagnosis of AMI, allowing earlier therapeutic interventions in AMI patients and the more rapid discharge of patients without AMI. Although clearly superior to earlier AMI biomarkers in detecting myocardial ischemia, cTn are far from ideal. The level of oxidation/reduction of the troponin molecule, phosphorylation, fibrin strands, and heterophilic antibodies have the potential to interfere with TnI assays [29]. Circulating troponin levels increase as early as 2–4 h after the onset of AMI, but still leave a “troponin gap” that prevents earlier detection of AMI, which could further improve prognosis in these high-risk patients. Finally, whereas the use of hs-cTn testing has massively increased diagnostic sensitivity, this has come at the cost of a decrease in diagnostic specificity. Indeed, several cardiac and non-cardiac factors (Table 2) have been shown to affect troponin levels [30]. Even mild increases in troponin levels, regardless of the underlying cause, have been shown to predict adverse prognoses. This makes troponin assessment in the emergency department an excellent test to reliably rule out low-risk individuals. However, based solely on troponin levels, many individuals will continue to undergo further evaluations, potentially delaying therapy for other, non-cardiac causes of troponin elevation [31]. Moreover, the high sensitivity of new generation hs-cTn assays could increase emergency department workload by blurring the line between health and disease (i.e., designating “healthy” individuals as being “ill”) and, by including such patients in the AMI category, could also lead to an apparent improvement in AMI outcomes [32].

**Figure 2:**
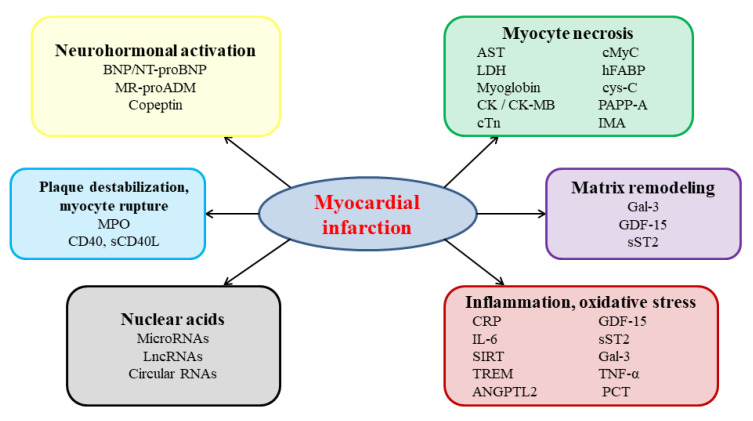
Pathophysiological pathways in acute myocardial infarction and examples of associated candidate circulating biomarkers; ANGPTL2—angiopoietin-like 2; BNP—B-type natriuretic peptide; CD40—cluster of differentiation 40; cMyC—cardiac myosin-binding protein C; CRP—C-reactive protein; cys-C—cystatin C; Gal-3—galectin-3; GDF-15—growth differentiation factor 15; hFABP—heart-type fatty acid binding protein; IL-6—interleukin 6; IMA—ischemia-modified albumin; LncRNAs—long non-coding ribonucleic acids; MPO—myeloperoxydase; MR-proADM—midregional proadrenomedullin; NT-proBNP—N-terminal fragment of the B-type natriuretic peptide precursor; PAPP-A—pregnancy-associated plasma protein-A; PCT—procalcitonin; RNA—ribonucleic acids; sCD40L—soluble ligand of cluster of differentiation 40; SIRT—sirtuins; sST2—soluble suppression of tumorigenicity factor 2; TNF-α—tumor necrosis factor α.

The continuous progress in the treatment of cardiac diseases and the introduction of new therapeutic approaches has imposed the rapid and continuous development of new laboratory assays [33]. The wide use of percutaneous coronary interventions, the introduction of newer generation antiplatelet agents, and recognition that in AMI, such strategies need to be applied as early as possible, have spurred the search of novel AMI biomarkers. These possible key tools can improve AMI diagnostic accuracy or enable the instant ruling out of AMI without the need for serial measurement (i.e., increase specificity) and enable faster detection of AMI (i.e., increase sensitivity). To accomplish these goals, novel biomarkers will have to meet two crucial criteria: accuracy and speed [34]. Any new AMI biomarker will therefore have to be specifically expressed in the myocardial tissue, at relatively high levels, and be released into the bloodstream rapidly after symptom onset. Several novel biomarkers that promise to fulfil at least part of these needs have been recently identified (Figure 2) [7,35,36,37,38].

### 4.1. Biomarkers of Myocardial Necrosis

Recent studies have supplemented the set of classic biomarkers of myocardial necrosis with a number of additional biomarkers, including the heart-type fatty acid binding protein (hFABP), the ischemia-modified albumin (IMA), and the sarcomeric cardiac myosin-binding protein C (cMyC).

Similarly to myoglobin, hFABP, a low-molecular-weight, non-enzymatic protein involved in the intracellular buffering and transport of long-chain fatty acids, is rapidly (i.e., within ≈3 h) released into the circulation after the onset of myocardial injury, its levels returning to baseline 12–24 h after the acute ischemic event [39]. Although hFABP appears to add incremental value to cTn, increasing diagnostic accuracy and accelerating clinical diagnostic decisions, hFABP is also expressed in the kidney and skeletal muscle, and its elimination is highly dependent on kidney function [40]. Thus, its value as a diagnostic marker of AMI remains controversial.

Acute myocardial ischemia induces major protein changes, including alterations of the N-terminus of albumin, leading to the formation of IMA. In the same manner to myoglobin and hFABP, IMA levels also increase rapidly (i.e., within 3 h) after AMI onset. A combined approach, using IMA and TnT at presentation for chest pain, appeared to increase AMI diagnostic accuracy compared with TnT alone [41]. However, when used alone, IMA displays sensitivity too low (i.e., 70%) to allow useful clinical decision-making, and its specificity is significantly affected by the fact that IMA levels also increase in a large variety of other medical conditions, as well as infections, liver, and advanced kidney diseases, cancer, or brain ischemia [42].

Sarcomeric cMyC is one of the most promising novel myocardial necrosis biomarkers. This myosin-binding protein isoform is exclusively expressed at the level of the heart, making it a specific marker of myocardial injury; it is more rapidly released into the bloodstream as a result of myocardial necrosis than troponin, allowing earlier detection of myocardial injury and disease [43]. The cardiac myosin-binding protein C myocardial concentration is almost twice that of cTn, which makes cMyC a more sensitive AMI biomarker than troponin, and the addition of cMyC to high-sensitive cardiac troponin T provides supplementary AMI-related diagnostic information [44].

Over the years, cardiovascular medicine has seen numerous, often major, paradigm changes. In atherosclerosis, concepts have moved from a purely lipid-related, to a more complex, lipid-inflammatory pathophysiology [45]. In atrial fibrillation, concepts have moved from a purely electrical disease to a more tangled combination of electrical, structural, autonomic, and molecular underlying changes [46,47,48]. In AMI, for years, biomarkers have been focused on the concept of myocardial necrosis. However, recent years have brought a major paradigm change in AMI biomarker research, and studies focusing on other ischemia-related mechanisms including neurohormonal activation, inflammation, plaque instability, and myocyte membrane rupture have started to emerge. With the extend of newer, more complex molecular techniques, multi-omic approaches have also started to be used for improvement of AMI diagnosis.

### 4.2. Biomarkers of Neurohormonal Activation

The observation that neurohormonal activation is a major change occurring in AMI patients has drawn attention to potentially novel AMI biomarkers, including the B-type natriuretic peptide (BNP) and the N-terminal fragment of its precursor (NT-proBNP), copeptin, and the midregional proadrenomedullin (MR-proADM).

Secreted by the ventricular myocytes in response to stretch, the proBNP precursor is cleaved into BNP and the inactive fragment NT-proBNP. Once released, BNP exerts vasodilating, natriuretic, hypocoagulative, inotropic, antiarrhythmic, and anti-renin–angiotensin–aldosterone and sympathoadrenal system effects, while promoting cell differentiation and tissue repair and supporting immunity, metabolic responses, and inflammation [49]. In patients with AMI, BNP and NT-proBNP levels have been associated with infarct size [50]. However, both BNP and NT-proBNP increase in a large variety of non-AMI-related settings, including some that may be accompanied by clinical symptoms comparable with AMI, such as acute heart failure and pulmonary embolism, as well as in kidney dysfunction, hypertension, chronic heart failure, myocarditis, cardiac arrhythmias, electrical cardioversion, or sepsis, precluding the use of these peptides as diagnostic AMI biomarkers, although they remain crucial for prognostic assessment in these patients [51]. According to 2020 ESC Guidelines for the management of acute coronary syndromes in patients presenting without persistent ST-segment elevation, the BNP and NT-proBNP plasma concentrations should be considered to contribute to patient’s prognosis (death, acute heart failure) [7].

Copeptin, a stable glycopeptide derived from the C-terminal fragment of the vasopressin prohormone, has been shown to be co-released with arginine vasopressin within the first 4 h after AMI. Circulating copeptin levels have been shown to exhibit linear correlation with those of arginine vasopressin, a key regulator of water homeostasis and plasma osmolality [52]. This makes copeptin an excellent surrogate marker of arginine vasopressin secretion, which has a very short half-life. When combined with cTn, copeptin has been shown to improve sensitivity and easily rules out AMI early [53]. However, copeptin is not a specific cardiac marker, neither is it recommended to routinely measure it for constant risk or prognosis judgement [7]. Other conditions, including kidney disease, heart failure, or sepsis, can also influence copeptin levels, and its levels have been shown to be affected by gender, body mass, hydration status, blood pressure, and glomerular filtration rate [51], thus precluding its use as a stand-alone diagnostic AMI biomarker.

Adrenomedullin is a stress hormone expressed in a large variety of tissues, including the brain, kidney, vasculature, and adrenal medulla, involved in diuresis and natriuresis, vasodilation, and inotropism regulation. Adrenomedullin is generated from its more stable precursor MR-proADM. In clinical studies, MR-proADM has been shown to significantly increase in AMI patients, particularly in those developing post-AMI heart failure, and high MR-proADM levels have been associated with significant increases in short- and long-term mortality and hospitalization for heart failure following AMI [54,55]. Increased MR-proADM levels have even been proposed as a rule-in criteria for AMI, but its exact value as a diagnostic biomarker in addition to the already established criteria remains to be evaluated.

Immediately after the onset of AMI, the renin–angiotensin–aldosterone system (RAAS) is activated, and this phenomenon is linked with an unfavorable prognosis [56]. High levels of angiotensin II aggravate myocardial ischemia-mediated vasoconstriction. In hypertensive women who experience an AMI, the renin–angiotensin–aldosterone axis is suggested to be upregulated [57].

Heightened RAAS activity demonstrates a determining implication not only within the pathogenesis of AMI, but also as an estimated model with 5 years post-AMI mortality, as revealed by the RAAS polymorphisms in the presence of AGT CC genotype and ACE allele, individual or in association [58].

Increased plasma renin activity (PRA) values were detected in both hypertensive and normotensive patients, in whom the diagnosis of AMI was ruled in; these were independently associated with a poor prognosis in AMI patients [59,60]. In the myocardium, increased aldosterone synthesis is induced by an acute coronary event, triggering ventricular fibrosis post-AMI [61].

### 4.3. Biomarkers of Inflammation

Myocardial inflammation is a critical process occurring in the setting of AMI that has been related to a variety of deleterious consequences, including electrical instability and increased risk of cardiac arrhythmias, autonomic dysfunction, and fibrosis development [48,62,63,64]. A wide variety of pro-inflammatory cytokines have been shown to increase in the setting of AMI and to predict prognosis in this population. This is particularly the case for C-reactive protein (CRP), interleukin- (IL-) 6, and procalcitonin (PCT).

Increased CRP levels have been linked with the extent of myocardial injury and with poorer prognosis in AMI patients. In animal studies, CRP removal was able to significantly reduce infarct size and improve outcomes [65]. Ries et al., in a pilot study in humans with AMI (CAMI-1 study), reported that CRP apheresis managed to reduce circulating CRP levels and a loss of correlation between CRP levels and infarct size, although CRP reduction was not associated with a significant reduction in infarct size [66]. However, because CRP increases in a wide variety of other settings, its levels are not sufficiently specific to qualify it as a reliable marker of AMI.

In the setting of AMI, increased IL-6 levels favor activation and recruitment of inflammatory cells, promote CRP release, and exert cardiac negative inotropic effects, predicting poor prognosis in this population [67]. Meanwhile, the IL-6 receptor blockade has been shown to reduce the inflammatory response and cTn release in patients with non-STE segment elevation AMI, suggesting that, similarly with CRP, IL-6 could emerge as not only a diagnostic AMI biomarker, but also as a potentially new therapeutic target in AMI [68]. However, the low specificity of IL-6 and the lack of large confirmatory studies limit IL-6 use as a diagnostic AMI biomarker.

The peptide precursor of the hormone calcitonin, PCT, has also been shown to increase in inflammatory settings, including AMI, and to have prognostic value in ischemic heart disease [69].

The potential role in the development of atherosclerosis and acute coronary syndromes of a group of zinc-dependent endopeptidases, namely, matrix metalloproteinases (MMPs), was investigated in previous studies [70,71,72].

The increased circulating levels of MMP 2, 3, 9 and 28 in AMI do not distinguish between atherothrombotic and non-atherothrombotic events. [70,73].

 Due to matrix metalloproteinase-9 linking with plaque rupture and myocardial necrosis, MMP-9 has demonstrated its position as an early-stage biomarker of AMI [70]. It was also suggested that the value of MMP-28 correlates with GRACE score and could be a predictor of short-term prognoses in AMI cases [72].

Other inflammatory biomarkers, including IL-1β, IL-37, and angiopoietin-like protein 2, have also been shown to increase in AMI patients, but further studies are needed before drawing a definitive conclusion in their regard [35].

### 4.4. Biomarkers of Plaque Destabilization and Myocyte Rupture

Myeloperoxidase (MPO), a component of neutrophil granules, is abundantly released from unstable atherosclerotic plaques and plays a critical role in myocardial inflammation and oxidative stress. When used alone, MPO does not seem to be appropriate for AMI diagnosis [74]. However, when combined with TnI and CK-MB, MPO appears to improve the early diagnostic accuracy of AMI [75].

Cluster of differentiation 40 protein, CD40, and its soluble ligand, sCD40L, are expressed by a wide variety of cell types and have been shown to be released during cardiac myocyte rupture. Both molecules are involved in inflammation and thrombosis and have been proposed as diagnostic AMI biomarkers [76]. However, their exact value remains to be established [35].

Biomarkers, including lipid markers (lipoprotein A, apolipoproteins A and B), endothelial cells- (endocan) and platelet- (mean platelet volume, mean platelet volume-to-platelet count ratio, beta-thromboglobulin, platelet miR-126) related markers have been proposed as AMI biomarkers [14,51]. Further enhanced studies are required to establish the additional value in AMI diagnosis for other biomarkers such as the degree of mobilization of mononuclear and endothelial progenitor cells, triggering receptors expressed on myeloid cells 1 and 4, cystatin C, pregnancy-associated plasma protein-A, class II phosphatidylinositol 3-phosphate kinase, sirtuins, arginine methyltransferase 5, and chitinase-3-like protein 1 [30].

The main characteristics of newer biomarkers potentially used in the diagnosis of AMI are depicted in Table 5.

Studies about other newer biomarkers, such as CRP, IL-6, procalcitonin, CD40, myeloperoxydase, and sCD40L, could be valuable in the diagnosis and prognosis of AMI, although further studies are needed [14,35]. Guidelines recommend the use of BNP/NTproBNP in the setting of different situations, especially in patients with suspected heart failure, as a prognostic indicator against AMI diagnosis biomarker and supporting the risk stratification of adverse outcomes [81].

## 5. A Glimpse into the Future—Emerging Molecular Candidate Biomarkers for Acute Myocardial Infarction

The development of new, high-technology laboratory methods continues to improve therapy monitoring, as well as diagnostic and prognostic accuracy in cardiovascular medicine [36,82]. With the advent of novel molecular biology techniques, evaluation of the entire molecular chain, from DNA to RNA, proteins, and metabolites has become possible. Multi-omic approaches based on genomic, transcriptomic, proteomic, and metabolomic studies that allow the evaluation of RNAs, peptides and proteins, and metabolites using whole blood, plasma, or serum, or even selected circulating cell types or extracellular vesicles, promise to be a game changer in AMI assessment.

### 5.1. Novel Peptide, Protein, and Enzyme Candidates for Acute Myocardial Infarction Diagnosis

Growth differentiation factor 15 (GDF-15), galectin-3 (Gal-3), and soluble suppression of tumorigenicity factor 2 (sST2) appear to be among the most promising novel AMI biomarkers and have been extensively studied over the past years.

Circulating levels of GDF-15, a member of the transforming growth factor beta cytokine superfamily expressed in a wide variety of cardiovascular and non-cardiovascular cells, have been shown to increase in inflammatory and high oxidative stress states and tissue injury, including AMI, and to predict prognosis in this population [83]. Galectin-3 has also been shown to participate in inflammation, cardiac fibrosis and repair, and maladaptive ventricular remodeling, as well as in atherosclerotic plaques formation, destabilization, and rupture. Increased Gal-3 levels have been reported in AMI patients, in whom Gal-3 has also been proposed as a valuable predictive marker [84]. Moreover, experimental data suggest that whereas Gal-3 could initially contribute to myocardial repair and the preservation of cardiac geometry and function, over the long term, Gal-3 activation could promote myocardial fibrosis and adverse ventricular remodeling [85]. Likewise, sST2, a member of the IL-1 receptor family, has been shown to increase in AMI patients and to predict prognosis in this setting [86]. However, due to insufficient specificity, sST2 is unlikely to enter clinical practice as a diagnostic AMI biomarker. Other potential peptide and protein biomarkers, including glycogen phosphorylase isoenzyme BB, involved in myocardial carbohydrate metabolism regulation and released rapidly after myocardial injury; S100A, a class of small-molecule calcium-binding proteins involved in cell division and metabolism regulation and highly sensitive in depicting myocardial injury; irisin, a recently identified hormone that facilitates glucose uptake and improves cardiac muscle activity; and adropin, a secretory protein involved in regulation of energy metabolism and insulin resistance, are currently under evaluation [14]. Several of them have been proposed as independent predictors of post-AMI outcomes, but none has demonstrated clear advantage over cTn in AMI diagnosis to date. 

### 5.2. Metabolomics—Emerging Targets for Acute Myocardial Infarction Diagnosis

By allowing rapid assessment of various products of cell metabolism, including during acute events, metabolomics has emerged as a promising new source of highly specific diagnostic AMI biomarkers. Studies in patients with septal ablation-induced AMI identified several myocardial cells metabolites released very early (i.e., within 10 min) after AMI onset [87]. These include molecules involved in the pentose phosphate, pyrimidine, and tricarboxylic acid pathways. However, the exact value of these new biomarkers in AMI diagnosis in addition to or instead of cTn remains to be established.

### 5.3. Circulating Ribonucleic Acids as Potential Diagnostic Biomarkers of Acute Myocardial Infarction

Non-coding RNAs, including microRNAs (miRNAs), circular RNAs, and long non-coding RNAs (lncRNAs), act as strong, tissue- and cell-specific epigenetic regulators of cardiac gene expression, homeostasis, and function, and have recently emerged as promising biomarkers in a wide variety of cardiovascular diseases. These molecules circulate in the peripheral blood, either bound to transport proteins or packaged in microparticles, thus being stably transported in the blood, protected from degradation, and detectable in blood samples. A number of these circulating non-coding RNAs have been or are currently under investigation as potential diagnostic AMI biomarkers.

MicroRNAs are small, single-stranded RNA molecules that work as post-transcriptional protein synthesis suppressors via gene silencing. Four miRNAs—miR-1, miR-133a/b, miR-208b, and miR-499, the latter two being expressed solely in the cardiac myocytes—have been consistently reported to increase in AMI patients, although controversies still remain regarding the value of individual miRNAs as AMI biomarkers [68]. With 78% sensitivity and 82% specificity, total miRNA levels appear to be suitable diagnostic biomarkers of AMI, with miR-499 appearing to be among the most significantly associated with AMI, increasing very early (i.e., within few hours after AMI onset), to very high (i.e., up to 3 × 10^5^) levels, and returning to baseline within hours or days after the acute event, while also reaching high (i.e., 88% and 87%) sensitivity and specificity [88]. Other miRNAs, including miR-21, miR-92a, miR-122, miR-181a, miR-320a, miR-328, and miR-375, have also been shown to increase in the peripheral blood in the setting of AMI [89]. Certain miRNAs appear to be highly specific for AMI and to display faster release kinetics than cTn. Moreover, studies have suggested that the miRNA signature could even distinguish between ST and non-ST segment elevation myocardial infarction [90]. In addition, in rats with experimental AMI, antagonizing certain miRNAs, such as miR-31, managed to reduce infarct size and post-AMI ventricular remodeling, preserving cardiac structure and function [91]. Several lncRNAs, namely, myocardial infarction-associated transcript (MIAT), H19, and metastasis-associated lung adenocarcinoma transcript 1 (MALAT1), have also been reported to be altered in AMI patients, but studies in this regard are still in their infancy [92,93,94,95].

## 6. Gaps in Knowledge and Future Research

The widespread use of cTn has revolutionized AMI diagnosis. Particularly, with the advent of hs-cTn, we can confidently say that sensitivity is no longer an issue in AMI diagnosis. However, two problems remain to be solved. Firstly, the increased sensitivity provided by hs-cTn assays came with a cost—an inevitable decrease in diagnostic specificity. Secondly, although cTn increase relatively early after the onset of AMI, they still leave a time gap between the onset of myocardial ischemia and our ability to detect it, thus precluding very early management of AMI. Historical markers, such as myoglobin, increase much faster than cTn, but they do not provide sufficient diagnostic specificity.

To date, all AMI biomarkers have focused on molecules released by the cardiac myocytes as a result of myocardial necrosis. More recently, understanding of the complex AMI pathophysiology has opened the way for new biomarker identification. Acknowledgment that processes such as inflammation, neurohormonal activation, or myocardial stress occur much earlier than myocyte necrosis has allowed us to expand biomarker research in these areas (Figure 1). Although most of the emerging biomarkers are not specific enough to impose as stand-alone AMI biomarkers, and are therefore unlikely to replace cTn in the foreseeable future, they could bring additional value for AMI diagnosis when included in multi-biomarker approaches. Including tissue-specific markers, such as certain miRNAs, could also be of invaluable help.

Multi-omic approaches hold great promise for more accurate and faster AMI diagnosis. MicroRNAs appear to be particularly promising in this regard, given that myocyte stress induced by anoxia, acidosis, and/or edema precedes myocardial necrosis in AMI patients and is rapidly reflected in circulating miRNAs levels. The rapid progress in molecular biomarker research must be matched, however, by similar progresses in laboratory techniques. Indeed, bioluminescence, solution-phase bioluminescence, and high-throughput sequencing methods have been developed for miRNA detection, but improvement is still necessary to provide instruments that can accurately, conveniently, inexpensively, and rapidly quantify circulating miRNAs in a large number of hospital laboratories. Integrated interpretation of multi-omics data also remains rather difficult at present, and may require machine learning to transform this approach into a clinically useful diagnostic tool.

Multi-biomarker strategies that reflect several AMI-related pathophysiological processes and include several enzymatic, non-enzymatic, and/or molecular markers may provide the solution for obtaining highly sensitive, highly specific, and very rapid AMI diagnoses. Such an approach was already shown to be superior to individual marker assessment for predicting post-AMI outcomes [96]. In a pilot study, a multi-marker panel termed “plaque disruption index” has been shown to present higher diagnostic accuracy for type 1 AMI than coronary angiography [97]. However, large clinical trials are still required before drawing definitive conclusions in this regard.

Regardless of future AMI diagnoses relying on single- or multi-marker approaches, any new approach will have to provide more readily available, more affordable, and more clinically relevant information than the strategies currently used in clinical practice, while also complying with high standards of precision and accuracy. Markers that do not fulfill these requirements will most likely not find their place in clinical practice. The ideal AMI biomarker, which is exclusively expressed in the myocardial tissue and whose plasma levels are not affected by any other pathology, remains to be identified. MicroRNAs could prove of invaluable help in this regard. It is unlikely that a single miRNA will prove superior to the traditional markers for AMI diagnosis. However, a combination of miRNAs, reflecting cardiomyocyte stress, inflammation, endothelial cells, and fibroblast damage, and/or other AMI-related pathophysiological processes, could change the future of AMI diagnosis. Moreover, miRNA-based therapies have the potential to modulate an entire pathway by regulating multiple genes at the same time. The advent of such therapies, and particularly of anti-miRNA strategies, is therefore expected to spur further research in this area.

The massive number of emerging biomarkers and the impressive progress achieved over the past decades in AMI diagnosis were spurred by a revolutionary paradigm shift—one that has drawn AMI diagnosis out of the ‘myocardial necrosis box’. Further understanding of AMI pathophysiology is expected to open new directions of research and enable the identification of even better diagnostic AMI biomarkers. Laboratory techniques will have to parallel this effort and ideally provide POCT systems able to detect single analytes as well as panels of circulating markers, and thus to enable accurate and rapid AMI diagnosis, including during patient transport to hospital. This would allow further cutting down time in AMI management, which in turn is expected to result in considerable improvement in AMI outcomes, both over the short and the long term.

## 7. Conclusions

Circulating biomarkers are key to AMI diagnosis. With their increased specificity, cTn have revolutionized the diagnosis, as well as the management of AMI. The advent of hs-cTn covered an unmet need and boosted diagnostic sensitivity, allowing more rapid therapeutic interventions. Unfortunately, this increased sensitivity was accompanied by a drop in specificity, not for the analyte, but for the diagnosis of AMI. Up to two-thirds of patients presenting with cTn levels that overtly exceed the threshold for AMI diagnosis do not have AMI and undergo costly, time-consuming, often invasive, although completely futile investigations. Further advancement in AMI diagnosis is therefore required. Traditional markers have all been focused on detecting myocardial necrosis. Acknowledgment that other processes, such as inflammation, neurohormonal activation, or myocardial stress, occur much earlier than myocyte necrosis, has opened the way for new approaches in AMI biomarker research. The ideal biomarker, that would allow rapid and reliable AMI rule in and rule out, is still to be discovered. However, biomarker research in AMI has massively advanced over the past years, and a solution to this problem is expected to be found in the near future, most likely in the form of multi-marker assessment, based on a combination of biomarkers that reflect several axes involved in the natural evolution of AMI. Future clinical trials, with large sample sizes, will have to confirm the utility of biomarkers that are currently under investigation, to identify novel potential biomarkers, and to validate the clinical impact of multi-biomarker-based AMI diagnosis.

## Figures and Tables

**Table 1:** Features of historical and current biomarkers used for acute myocardial infarction diagnosis.

Biomarker	TFPT (h)	TPL (h)	TRB	Sensitivity (%) *	Specificity (%) *	PPV (%) *	NPV (%) *
AST	12–24	24–48	10–14 days	75	71	75	71
LDH	6–12	24–72	8–14 days	82	70	76	77
Myoglobin	0.5–2	6–12	12–24 h	79	89	98	60
CK	3–8	12–24	48–72 h	95	68	30	99
CK-MB	4–8	12–24	48–72 h	92	90	98	83
cTn	3–6	10-24	5–10 days (TnI) 10–14 days (TnT)	97–100	94–97	98–99	88–100

TFPT—time to first positive test; h—hours; TPL—time to peak levels; TRB—time to return to baseline PPV—positive predictive value; NPV—negative predictive value; AST—aspartate aminotransferase; CK—creatine kinase; CK-MB—creatine kinase-myocardial band; cTn—cardiac troponins (i.e., T and I); LDH—lactate dehydrogenase. * Data reflect values obtained for serial measurement.

**Table 2:** Most common non-acute myocardial infarction-related causes of cardiac biomarkers elevation.

Biomarker	Potential Causes of Elevation (Others Than Acute Myocardial Infarction)
AST	Liver diseases (hepatitis, cirrhosis, carcinoma, liver necrosis, cholestasis); Skeletal muscle injury (trauma, myopathy); Hemolysis; Infectious mononucleosis; Shock, sepsis.
LDH	Hemolytic anemia, hemolysis; Liver diseases (hepatitis, cirrhosis, carcinoma, liver necrosis, cholestasis); Stroke; Pancreatitis; Skeletal muscle injury (exhaustive exercise, muscle trauma, rhabdomyolysis, muscular dystrophy, polymyositis, alcohol myopathy, seizures); Carcinomas, leukemia; Hypothyroidism; Lung diseases; Shock, sepsis.
Myoglobin	Skeletal muscle injury (exhaustive exercise, muscle trauma, rhabdomyolysis, muscular dystrophy, polymyositis, alcohol myopathy); Surgery; Shock, sepsis, burns; Chronic kidney disease; Carcinomas (colon, lung, prostate, endometrium).
CK-MB	Significant skeletal muscle injury (trauma, rhabdomyolysis, convulsions, muscular dystrophy, intramuscular injections); Cocaine abuse; Shock, sepsis; Malignancies; Hypothyroidism; Heart conditions (heart failure, myocarditis/pericarditis, aortic dissection, cardiac arrhythmias, cardiac trauma, cardiac surgery, cardioversion, cardiomyopathies, cardiotoxic drugs); Chronic kidney disease.
cTn	Heart conditions (heart failure, myocarditis/pericarditis, aortic dissection, cardiac arrhythmias, cardiac trauma, cardiac surgery, cardioversion, cardiomyopathies, cardiotoxic drugs); Lung diseases (pulmonary embolism, severe pulmonary hypertension, chronic obstructive pulmonary disease); Chronic kidney disease; Significant skeletal muscle injury (trauma, rhabdomyolysis); Sepsis; Systemic inflammatory diseases.

AST—aspartate aminotransferase; CK-MB—creatine kinase-myocardial band; cTn—cardiac troponins (i.e., T and I); LDH—lactate dehydrogenase.

**Table 3:** Troponin T assay specific cut-off levels in the rule-in or rule-out 0 h/1 h and 0 h/2 h algorithms in NSTEMI diagnosis; values are expressed in ng/L; adapted from [7].

Variation	Elecsys^®^ Troponin T high-Sensitive Assay (Roche Diagnostics)	
	0 h/1 h	0 h/2 h
Very low	<5	<5
Low	<12	<14
No hΔ	<3	<4
High	≥52	≥52
hΔ	≥5	≥10

**Table 4:** Troponin I assay specific cut-off levels in the rule-in or rule-out 0 h/1 h and 0 h/2 h algorithms in NSTEMI diagnosis; values are expressed in ng/l; adapted from [7].

	Assay/Manufacturer													
Variation	Architect/ Abbott		Centaur/ Siemens		Access/ Beckman Coulter		Clarity/ Singulex		Vitros/Clinical Diagnostics		Pathfast/ LSI Medience		TriageTrue/ Quidel	
	0 h/1 h	0 h/2 h	0 h/1 h	0 h/2 h	0 h/1 h	0 h/2 h	0 h/1 h	0 h/2 h	0 h/1 h	0 h/2 h	0 h/1 h	0 h/2 h	0 h/1 h	0 h/2 h
Very low	<4	<4	<3	<3	<4	<4	<1	<1	<1	<1	<3	<3	<4	<4
Low	<5	<6	<6	<8	<5	<5	<2	*TBD*	<2	*TBD*	<4	*TBD*	<5	*TBD*
No hΔ	<2	<2	<3	<7	<4	<5	<1	*TBD*	<1	*TBD*	<3	*TBD*	<3	*TBD*
High	≥64	≥64	≥120	≥120	≥50	≥50	≥30	≥30	≥40	≥40	≥90	≥60	≥60	≥60
hΔ	≥6	≥15	≥12	≥20	≥15	≥20	≥6	*TBD*	≥4	*TBD*	≥20	TBD	≥8	*TBD*

**Table 5:** Characteristics of newer biomarkers used in the diagnosis of acute myocardial infarction.

Biomarker.	Value	TFPT (h)	TPL (h)	TRB (h)	Sensitivity (%)	Specificity (%)	PPV (%)	NPV (%)	References
hFABP	7 µg/L (cut-off)	3 h	-	12–14	81.8%	100.0%	55%	66.7%	[35,77]
IMA	88.2–111.8 U/mL (reference interval)	3 h	6	24	70%	80%	96%	91%	[78]
cMyC	10 ng/L	<60 min	2	-	100%	41.3%	27.3%	100%	[79]
Copeptin	2.18–2.35 ng/mL reference interval	0–1 h	0–1 h	12–36 h	79.41–87.80%	60.38–62.73%	40.91–46.15%	89.93–98.17 %	[53,80]

TFPT—time to first positive test; h—hours; TPL—time to peak levels; TRB—time to return to baseline PPV—positive predictive value; NPV—negative predictive value.
